# Assisted damage closure and healing in soft robots by shape memory alloy wires

**DOI:** 10.1038/s41598-023-35943-6

**Published:** 2023-05-31

**Authors:** Seyedreza Kashef Tabrizian, Seppe Terryn, Aleix Costa Cornellà, Joost Brancart, Julie Legrand, Guy Van Assche, Bram Vanderborght

**Affiliations:** 1 Brubotics, Vrije Universiteit Brussel (VUB) and Imec, Brussels, Belgium; 2 Physical Chemistry and Polymer Science (FYSC), Vrije Universiteit Brussel (VUB), Brussels, Belgium

**Keywords:** Mechanical engineering, Polymers

## Abstract

Self-healing soft robots show enormous potential to recover functional performance after healing the damages. However, healing in these systems is limited by the recontact of the fracture surfaces. This paper presents for the first time a shape memory alloy (SMA) wire-reinforced soft bending actuator made out of a castor oil-based self-healing polymer, with the incorporated ability to recover from large incisions via shape memory assisted healing. The integrated SMA wires serve three major purposes; (i) Large incisions are closed by contraction of the current-activated SMA wires that are integrated into the chamber. These pull the fracture surfaces into contact, enabling the healing. (ii) The heat generated during the activation of the SMA wires is synergistically exploited for accelerating the healing. (iii) Lastly, during pneumatic actuation, the wires constrain radial expansion and one-side longitudinal extension of the soft chamber, effectuating the desired actuator bending motion. This novel approach of healing is studied via mechanical and ultrasound tests on the specimen level, as well as via bending characterization of the pneumatic robot in multiple damage healing cycles. This technology allows soft robots to become more independent in terms of their self-healing capabilities from human intervention.

## Introduction

**Figure 1:**
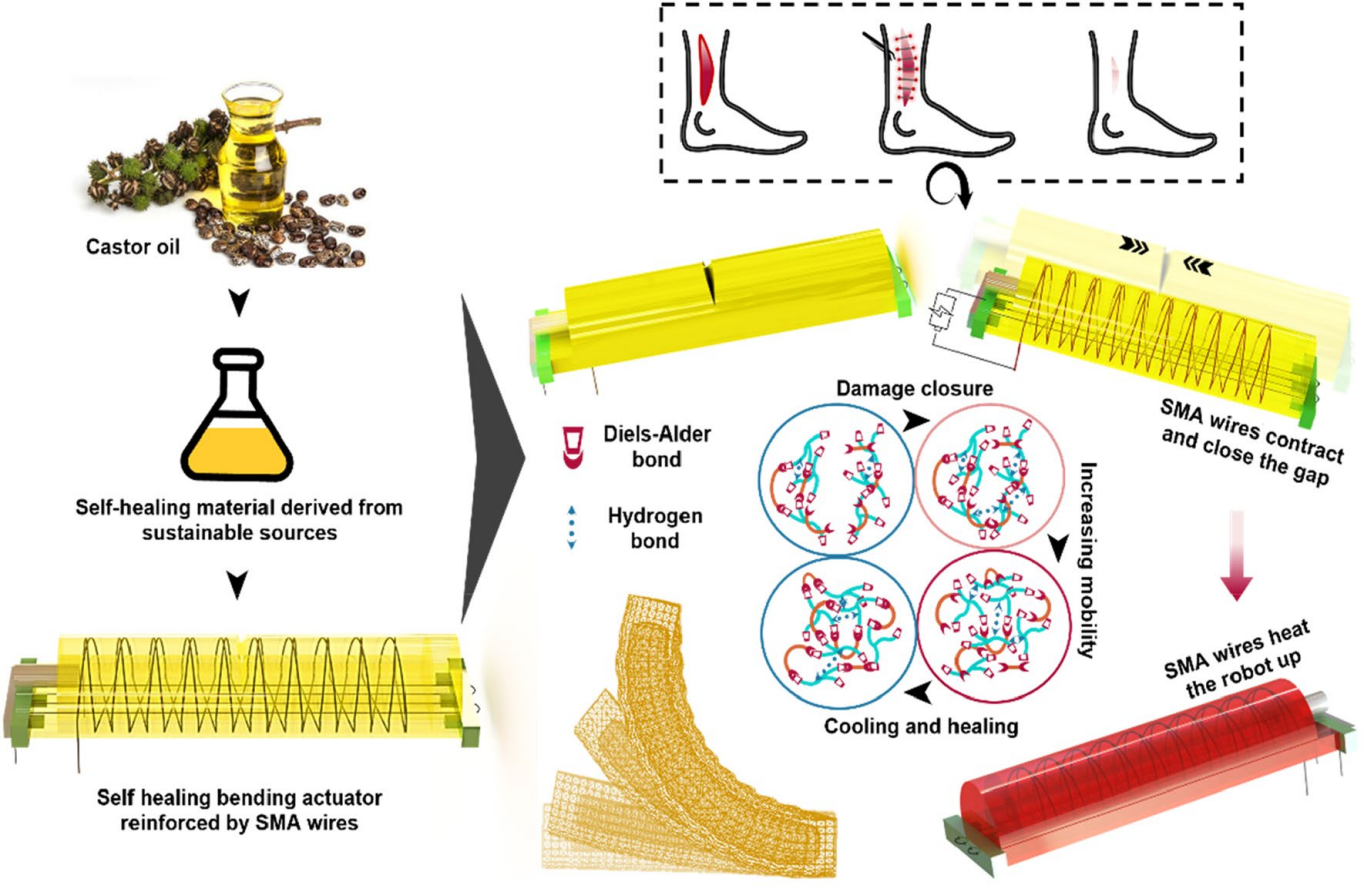
Analogy between human body and the soft robot in recovering from large incisions. It is made out of a castor oil based self-healing elastomer. After closing the incision in the actuator, the SMA wires keep activation for heating the robot up and accelerating the healing.

To address the vulnerability of soft robotics, self-healing and damage resilient materials have recently been introduced in the field^1–5^. Damage healing in soft robots is limited to recombining fracture surfaces that are brought back in contact on a microscopic level^2^. For relatively small damage in elastomers, the elastic response of the material reintroduces the fracture surfaces into contact, allowing efficient healing^6^. However, in the dynamic, unstructured environment where these soft robots will operate, they will be exposed to events that may lead to large cuts, tears and bursts. In these cases, the healing is not possible without bringing both damage surfaces into contact. This reconnection is essential for the effective bond (re)formation across the damage surfaces to recover the mechanical properties and other functions (Fig. 1).

Although healing of large damages in soft robots has been proven in the previous work^2^, its success relied on multiple external manual interventions, including observation/detection of the damage, taking the robot to a rest position (removing the pressure in pneumatically driven ones), manually closing the gap in case of large damages, and increasing the temperature to trigger the healing process. The latter was automated in our previous work through a healable heater^7^, embedded in a soft bending actuator, allowing to autonomously trigger the healing via joule heating. In addition, researchers have demonstrated different principles to autonomously detect and also localize the damage e.g., optical sensing technology^8^, electronic skin^9^. Nevertheless, the manual intervention for recombination and recontact after large damages is still required. To make soft robotics a truly resilient and sustainable solution in environments outside factories, healing should become completely autonomous.

This limitation is most pronounced in intrinsic self-healing materials, in which the healing capacity relies on chemical groups inherent to the material matrix^10^, e.g., reversible (physico)chemical bonds. Contrary, extrinsic self-healing materials contain liquid healing agents that are stored in reservoirs and can fill the damage volume as the reservoir ruptures, similar to the cardiovascular system in the human body^11,12^. However, the amount of healing agent that is stored in the reservoirs and is released upon damage is limited^13^. Consequently, current self-healing materials and structures require the application of an external force to close the large damage volumes before the healing process. This is similar to the human body (Fig. 1). While the body is capable of growing soft tissue and repairing wounds, which is similar to other creatures such as planarians^14^ and corals^15^, external interventions (e.g., stitches) are typically required for deep, gaping wounds or those located in areas with high tension or movement, such as the face or joints.

Material-based approaches have been developed that can provide damage closure prior to healing. Inspired by the regenerative power in nature, White et al*.* developed a two-stage extrinsic healing system: a shape-conforming dynamic gel fills the damage volume and a second reactive system polymerizes in the scaffold to restore the initial material properties^16^. Arguably, this regenerative potential even surpasses that seen in natural organisms. Similarly, researchers have developed different mechanisms to provide damage closure, generically referred to as “Close-Then-Heal” (CTH) that are unprecedented in natural systems^17^. Cerdan et al*.* have created a healing magnetic composite that is able to close wide gaps by the application of an external magnetic field using a permanent magnet^18^. Alternatively, the shape memory effect of polymers or metals propose great potential for temperature activated damage closure. Shape memory polymers (SMP) show the ability to close scratches and assist the healing^19–22^. Zhang and Li have reported a CTH system by embedding polymer artificial muscles in a thermoset matrix^23^. However, in this SMP assisted healing, heat has to be provided externally or by an integrated heater, resulting in a complex system with multiple integrated components.

Shape memory alloys (SMAs) on the other hand, are able to restore their predefined shape by heating them up to a certain temperature, externally or via joule heating through applying a current. The latter would simplify the complexity of the CTH system. SMAs are widely used in soft robotics for actuation due to the high-power to weight ratio^24–26^. Nevertheless, the problem of limited stroke and low actuation frequency have always been challenges for engineers^27^, although solutions are being researched for those limitations. Lee et al. have proposed free-sliding SMA wires instead of tendons in tendon-driven robots to increase bending angle^28^. Huang et al. have made an SMA-activated soft-bodied robot out of thermally conductive elastomers to speed up the frequency of motion^29^. In contrast, SMA wires are a viable solution for damage closure, as this is a relatively slow process requiring a limited stroke.

To the best of the authors’ knowledge, only few studies have been conducted on assistive healing by shape memory wires and are limited to specimen level, yet to be integrated in applications such as soft robots^30^. Most of them are in the domain of extrinsic healing materials where SMA wires and healing agents fill the gap synergistically^31–34^. Cohades et al*.* have stitched SMA wires in a glass fibre-reinforced intrinsic healable plate to close longitudinal cracks^35^. Kirkby et al*.* have also reported that the activation heat of the SMA wires can assist polymerization of the healing agent in an extrinsic self-healing system^34^. Saeedi and Shokrieh have assessed the effect of passive and active presence of the SMA wires in a self-healing resin and to what extent the activation force of SMA wires can increase the toughness of a double cleavage–drilled compression (DCDC) specimen^36^.

In this paper, SMA wires are exploited for both damage closure and as a heat-providing system in a self-healing robotic application (Fig. 1). Furthermore, as the third functionality, they are used as reinforcement to make the actuator bend in a preferential direction^37–39^. This is the first time that SMA wires are used with this high degree of multifunctionality, synergistically enabling the large damage closing/healing, healing rate and the actuation motion, while reducing the number of components in the system and consequently its complexity (Movie S1). To demonstrate the strength of this approach, several bending and tensile tests have been conducted to characterize the healing performance of the healable specimens with embedded SMA wires. In addition, an ultrasonic pulse analyzer is employed to characterize the closure of the incision. Finally, the functionality of the SMA wire-reinforced bending actuator is compared before and after damage-healing cycles. This overcomes two obstacles on the road of a sustainable future; (i) solving the problem of ecological footprint and longevity of soft robots, and (ii) as such paves the way for soft robots for real applications out of factories where human have limited access to.

## Materials and methods

### Bio-based sustainable material

In this work, we used our previously reported castor oil-based self-healing polymers with autonomous (healing at room temperature), yet heat-induced accelerated healing abilities^1^. Using these bio-based self-healing polymers, is a big step towards sustainable development^40–43^, as we decreased environmental impact on the following five levels^1^; (1) the polymeric network is based on raw feedstocks, rather than on fossils, (2) the one-pot synthesis is solventless, (3) these polymers recover from microscopic damage by self-healing, (4) they are completely recyclable, and (5) at their end of life, the materials are fully degradable. The secret of their fast and efficient low-temperature healing, in combination with their high mechanical strength and stability, is the synergy of weak hydrogen bond interactions and dynamic Diels–Alder covalent bonds (Fig. 1). Upon damage, the weak hydrogen bonds kickstart fast reconnection at the damage surfaces, resulting in the immediate recovery of an important fraction of the mechanical properties. The Diels–Alder bonds are the main contributors to the mechanical properties and prevent, or reduce, creep, yet take longer times to be fully restored^1^. These dynamic bonds were introduced by functionalizing castor oil fatty acids with furan functional groups in a one-pot, two-step synthesis. Two renewable cyclic anhydrides, maleic anhydride and itaconic anhydride were used to make two different materials with different mechanical properties. The functionalized castor oil with each of the anhydrides was then reacted with furfuryl glycidyl ether, which was derived from furfural, one of the most promising compounds for the synthesis of sustainable monomers^44^. The furan functional groups were crosslinked using a liquid, fatty acid-derived bismaleimide (BMI-689) at room temperature where castor oil serves as the backbone of the network. All steps were performed without using any solvents nor catalysts. The Young’s modulus of the maleic-based material is 1 MPa (hereafter referred to the stiff material) whereas the itaconic-based material has a modulus of 0.5 MPa (hereafter referred to the soft material) (Sect. S1).

As a generic support mechanism, the presented approach can be used for any self-healing polymer. None of the added values of the embedded SMA wires, including damage closure, stimulation and reinforcement, relies on the type of the self-healing material. Here, for specimen-level studies the stiff material was used, whereas the robot was made of the soft material to achieve more bending angle.

### Methodology of damage closure and healing in SMA-reinforced specimens

The wires used in the specimens are from the category of low temperature wire of Dynalloy with 0.2 mm in diameter and start activation temperature of 68 °C. They have been pre-strained by 4% upon delivery. The datasheet of the SMA wires is in Table S1.

To investigate the influence of self-activated closure, Joule heating and healing through current flow through the SMAs, one specimen was healed in an oven (Movie S2) and another via the Joule effect (Movie S3) of the wires. They were heated up to 75 °C—80 °C for 45 min and kept at room temperature for one day. Inside the specimens heated via Joule effect, 3 SMA wires were embedded with a 4 mm distance in between for a better heat distribution, while two wires sufficed for damage closure and heating/healing using an oven (Fig. S1). Based on the data in Table S1, for full activation of the 0.2 mm SMA wires, almost 600 mA was needed which could provide the desired temperature of 75–80 °C in the specimens. A FLIR One Pro thermal imaging camera was used to record joule-effect heating of the specimens. In each cycle, the specimens were manually cut in two using a scalpel blade and the parts are forced apart, resulting in a gap of more than 1 mm (Movie S4). As the wires were clamped in the specimens, the created gap equals the elongation of the SMA wires. The SMA wires were pre-strained to 4% by the manufacturer, and consequently upon a first damage healing through heating, the wires contracted, leading to closure of the damage. It is recommended not to overstrain the SMA wires more than 8% as they will lose their functionality much sooner than working in the safe range (below 8% of strain) where they can operate for millions of cycles. Therefore, based on the length of the wires and the specimens (Fig. S1), gaps as large as 2 mm can be closed.

### Ultrasound pulse analyzer

We used PULSONIC 58-E4900 and read the data with a frequency of 1 Hz. The position of the probes was fixed in all the experiments. Moreover, due to the softness of the specimen, it was placed on a manual lift platform to adjust the height and as such keeping constant the contact pressure between it and the probes in all experiments (Fig. S2). Clearly, it was necessary to have the damage between the probes.

### Principle of autonomous closing and healing of large gaping damages

**Figure 2:**
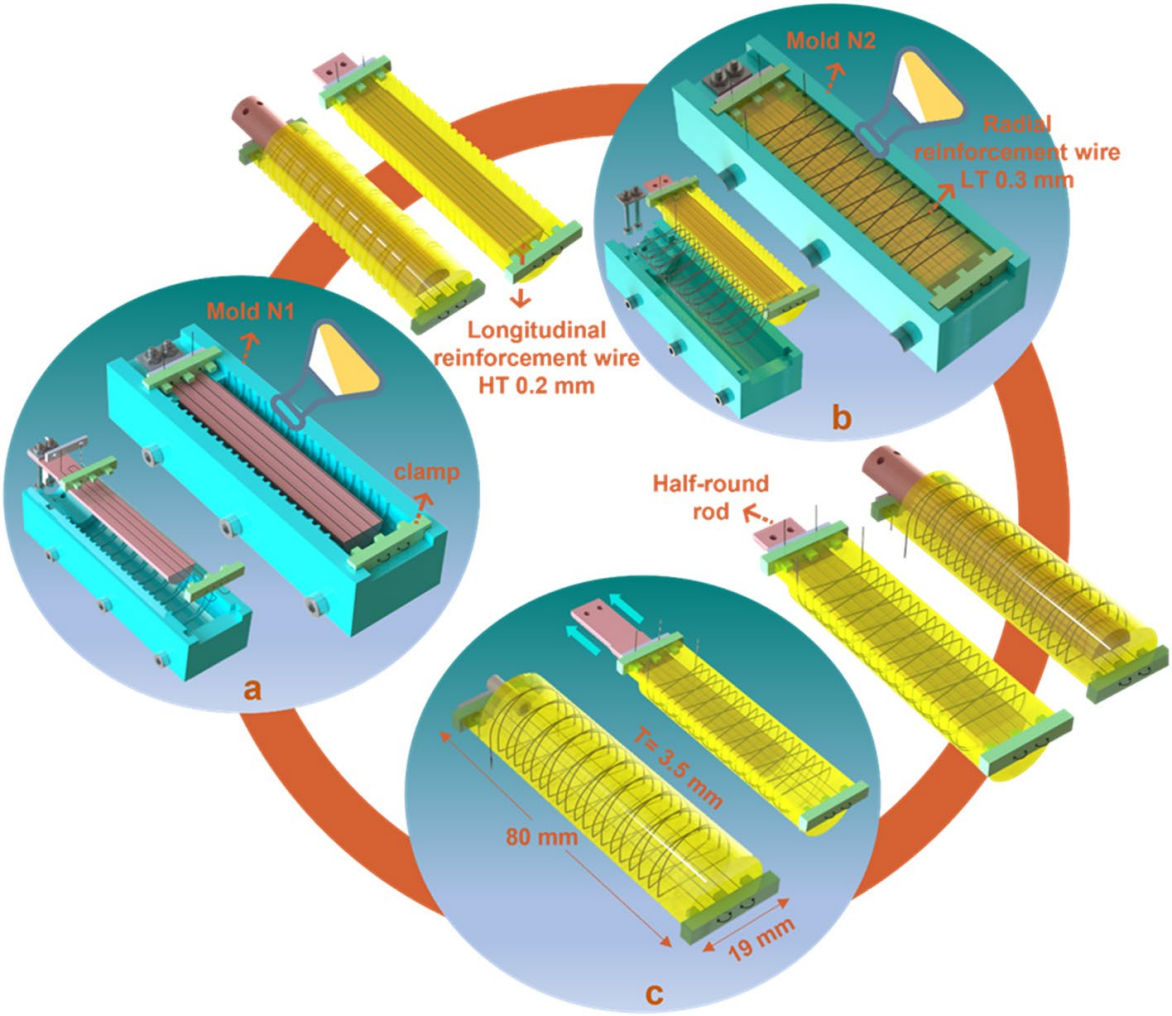
Processing of the SMA bending actuator. Soft chamber (**a**) longitudinally and (**b**) radially reinforced by SMA wires in a two-step molding/casting process followed by **(c)** attachment of the inlet via local heating/healing of the self-healing material in the third step.

Made out of the soft material^1^, an SMA wire-reinforced self-healing soft bending actuator was developed (Fig. 1). The bending performance was first evaluated by finite element analysis (Figs. S3, S4)^38,45^, and then the actuator was made in three steps, as seen in Fig. 2. First, the longitudinal SMA wire is integrated in the soft matter. Second, the radial SMA wire reinforces the chamber and is embedded inside it, followed by the inlet connection as the last step (Fig. 2). More information about the processing of the actuator is available in Sect. S5.

**Figure 3:**
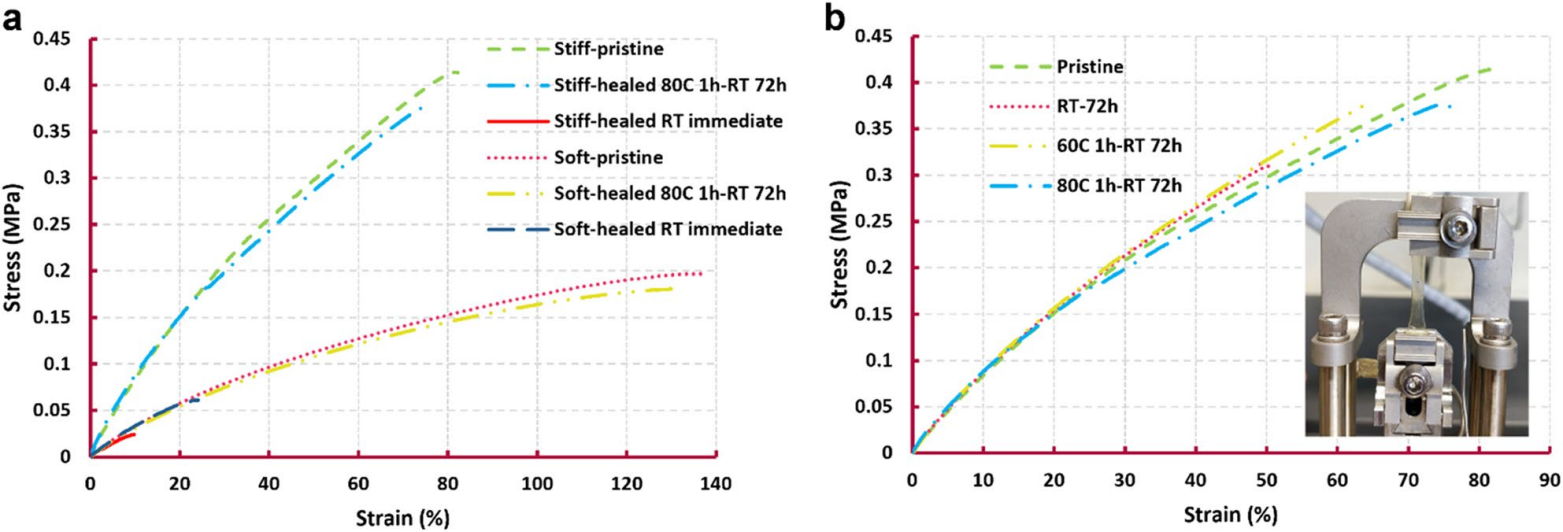
Mechanical characterization of the healable matrix. (**a**) Tensile test of the stiff material (maleic-based) and the soft material (itaconic-based) in initial state and healed state at 75–80 °C with 1% per seconds of strain rate. After heating the samples for an hour, they were left at room temperature for 3 days. In short time scale healing test, the samples were cut in two, realigned and immediately subjected to the tensile test (**b**) Tensile data of the stiff material under various healing profiles.

Looking at biology, deep and large wounds can be healed upon stitching, stapling or gluing. Similarly, when a large incision, cut or gap occurs in self-healing soft robots, the first healing requirement is to reconnect the fracture surfaces on a microscopic level. In this actuator, the integrated SMA wires take the responsibility of damage closure. Upon activation by passing an electrical current, the wires are contracted. This contraction is transferred to the soft chamber in which they are embedded, resulting in the closure of the damage (Fig. 1). Upon adequate recontact, the system is able to fully recover from this large damage as the material is capable of healing at room temperature^1,6^. However, room temperature healing usually takes time and full recovery is achieved only on the order of days (Fig. 3)^6,46^. Heating accelerates the process of healing by increasing simultaneously the mobility and the reactivity of the polymer network^46^. This is a fact that is seen in many self-healing materials^10^. The electrical current required to activate the SMA wires produces Joule heating. Being embedded in the chamber, the heat of the wires is well transferred to it (Fig. 1). Recontact is further enhanced on the microscopic level through this increased mobility at higher temperatures, while the increase in reactivity increases the rate of re-bonding across the joined fracture surfaces^1,46,47^. This noticeably decreases the healing time (79% conversion is reached after 150 min at 70 °C i.e. 33% at room temperature, which is 2.5 times faster, and 2.5 times more time to reach 90% efficiency in healing in case of room temperature healing^1^) and increases the healing efficiency (almost 1.5 times), meaning that a higher fraction of the mechanical properties of the system is recovered. Upon cooling, the polymer recovers its initial mechanical properties and the system is healed from a large gaping damage and recovers full functionality, without any human intervention.

It is generally accepted that the healing time increases with increasing size of the damage and varies for different types of damage (e.g. microcracks versus macroscopic damage). This is a physical phenomenon, since for the same material, the reaction kinetics do not care about the size of the damage^46^. The reaction kinetics depends only on the concentration of the reactive groups (broken bonds) and relatively the bond (re)formation is equally fast. Our method overcomes the physical and topological restrictions of the healing process. As such, upon excellent contact, which is the case when using SMAs (later is seen by ultrasound test), the healing time is independent of the damage size and type.

## Results

### Self-healing material

Figure 3 shows the mechanical properties of both the stiff and soft materials, derived through uniaxial tensile testing (Sect. S1). For healing tests, the samples were cut in two, manually recontacted and healed under different temperatures using an oven and subsequently tested in the tensile test. Figure 3a compares the response of the healed and pristine samples of both materials to tensile test. Considering fracture stresses and strains, more than 90% of the functionality is restored after heating at 70–80 °C for an hour, followed by remaining at room temperature for three days to complete the reaction. This efficiency is amongst the higher values reported in literature^5,10^. Furthermore, the healing performance of both the materials were studied at a short time scale, where tensile testing specimens were cut in two, realigned and contacted, and immediately tested in tension. As a result, it was possible to study almost the pure contribution of hydrogen bonds in the material's recovery at the short time scale (Fig. 3a). This can also be referred to as the surface adhesion analysis of the materials. It is worth noting that the formation of hydrogen bonds is much faster than Diels–Alder bonds^1^. The stiff material shows almost no recovery as the interface partially opened upon mounting in the tensile machine. This is reflected in the experimental results where the Young modulus of the stiff material in the short time scale healing test (red plot) does not recover to that of the pristine material (green plot), as shown by the slope of the lines. On the other hand, the soft material demonstrates a better short time scale healing property. The material's Young modulus is fully recovered, as evidenced by the comparison of the dark blue plot with the pink plot. Moreover, upon healing, the material recovers 20% and 33% of its maximum strain and stress, respectively, as determined by the strain and stress fracture points.

To clarify the role of heating during healing, Fig. 3b compares a pristine and three healed samples with different healing profiles. Based on the fracture stresses and strains, the non-heated sample shows almost 20% and 30% less healing efficiencies compared to the heated specimen at 80 °C, respectively. In addition, the one heated at 80 °C shows a better performance than the heated one at 60 °C. As mentioned, heating increases the mobility and reactivity of the polymer network and consequently results in a more efficient and accelerated healing^1,46^. For information about the effect of the increased temperature on the reactivity of the polymer network, the readers are referred to material-based studies and kinetic analysis of the network^1^.

### Close-then-heal analysis on the SMA reinforced specimens by mechanical tests

**Figure 4:**
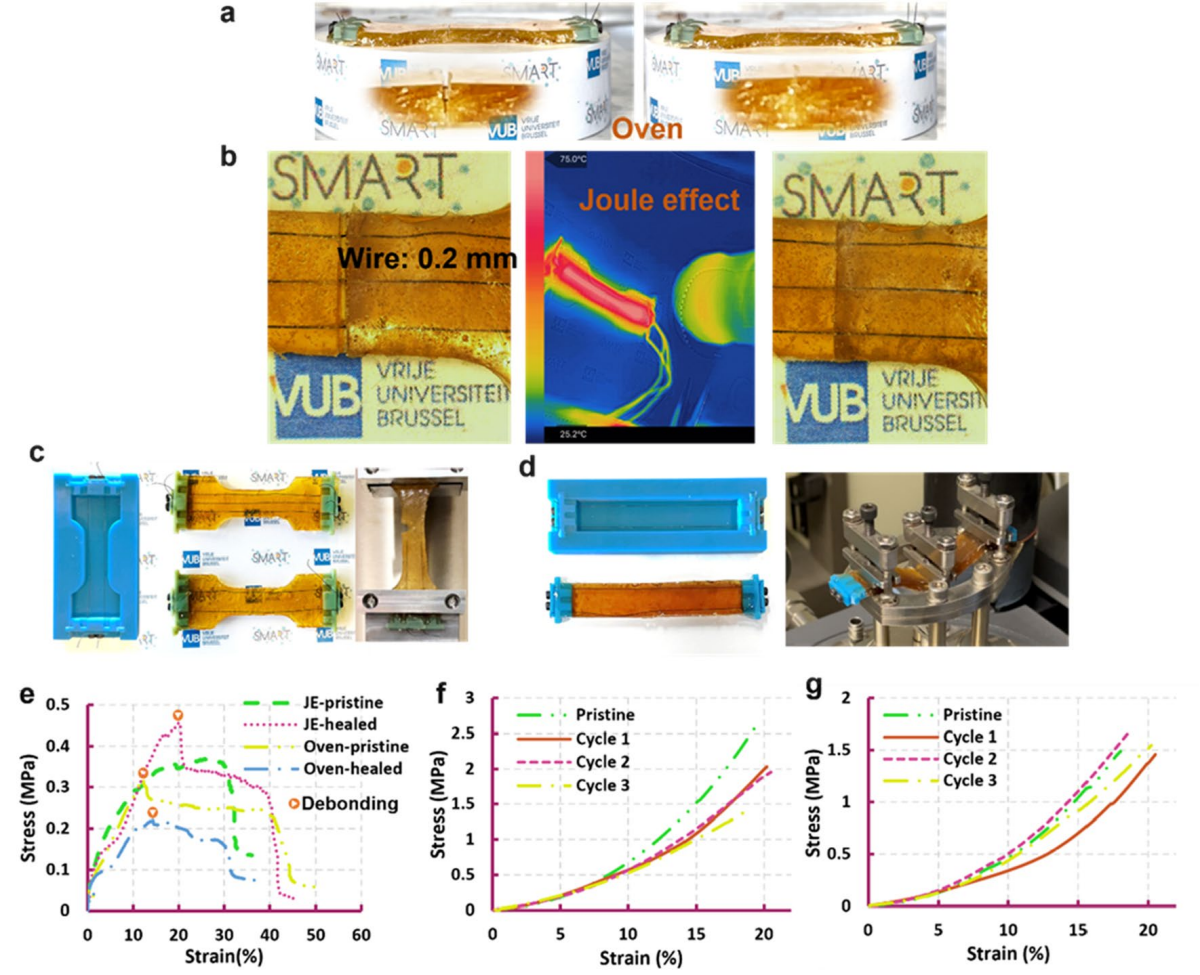
Close-then-heal characterization in SMA-reinforced specimens under tensile and bending tests. The specimens where heated up for 45 min and kept at room temperature for one day. (**a**) Damage closure of a sample heated in an oven by SMA wires. (**b**) Damage closure and heating a sample up by Joule-effect of the SMA wires. (**c**) Dog bone specimens in tensile test. (**d**) Rectangular specimens in bending test. (**e**) Tensile test of two closed then healed specimens heated by SMA wires’ Joule effect and in an oven at 75 °C for 45 min, in comparison with the pristine ones. (**f**) Three bending tests of a specimen closed and healed by three SMA wires. (**g**) Three bending tests of a specimen closed by two SMA wires and heated/healed by an oven.

The closed and healed samples heated via oven (Fig. 4a) and Joule-effect of the SMA wires (Fig. 4b), as well as the pristine ones are subjected to mechanical tests by which the healing performance is evaluated (Sect. S6) (Fig. 4c,d). In tensile testing (Fig. 4e) (Movie S5), the specimens break at lower fracture strains than the non-reinforced ones (Fig. 2a), while reaching almost the same level of stress. Debonding between wires and matrix in the composites is observed in three out of the four experiments before fracture of the specimens (Fig. 4e). Reinforced with 3 wires, the Joule effect-healed sample reaches higher stress in comparison with the one reinforced with 2 wires. The healing efficiency of both specimens is higher than 75%, considering the fracture stresses and strains. In Fig. 4f,g the healing efficiency of the specimens are measured in 3 cycles of bending tests where they are healed by Joule-effect and in the oven, respectively (Movie S6). All samples show excellent recovery of the mechanical behavior up to about 10% strain and reach the same fracture strain after healing. In terms of fracture stress, the healing efficiency of Joule effect-healed samples starts from 80% in the first and second cycles and drops to 60% in the third one (Fig. 4f). The specimen heated in the oven shows a slight loss of mechanical properties in the first healing cycle, which is completely recovered in the next two cycles (Fig. 4g).

### Damage closure analysis by ultrasonic test

**Figure 5:**
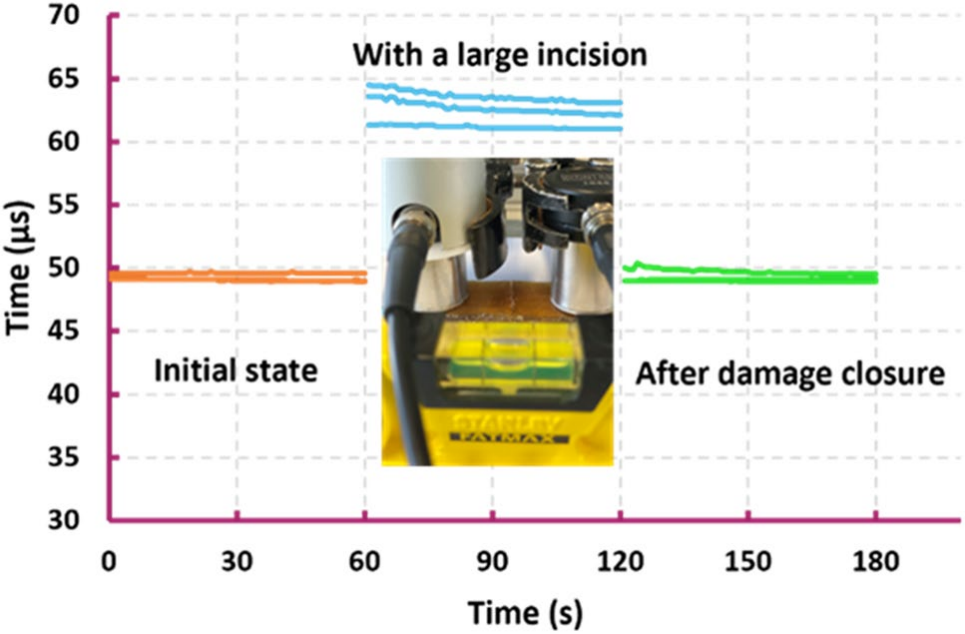
Ultrasound assessment of damage closure. With a created damage in the specimen, pulse transfer from the emitter probe to the receiver one takes longer.

Ultrasonic pulse analyzer is a device used for non-destructive health monitoring and is often used to detect damage in concrete^48^. It has two probes and measures the velocity of pulses transferred from the emitter probe to the receiver one through a medium in between (Fig. S2). While a crack is present in the medium, it can be detected by observing a change in the velocity of the transferred pulse. Here, we employ this fast and easy technique to demonstrate how efficient a large gap is closed in a specimen using our shape memory assistive closure approach. Three sets of measurements are done, on an undamaged, a damaged and a closed specimen. In each set, the test is repeated three times, ensuring the consistency and reproducibility of the data. As seen in Fig. 5, in an undamaged sample it takes almost 50 microseconds for the pulse to transfer between the probes. With damage in between, it takes more than 60 microseconds as the gap delays the pulse transfer. After activation of the SMA wires, the travel time of the pulse is again reduced to 50 microseconds, confirming the microscopic closing of the gap by means of shape memory assisted closure.

### Damage closure and healing of the SMA reinforced actuator

**Figure 6:**
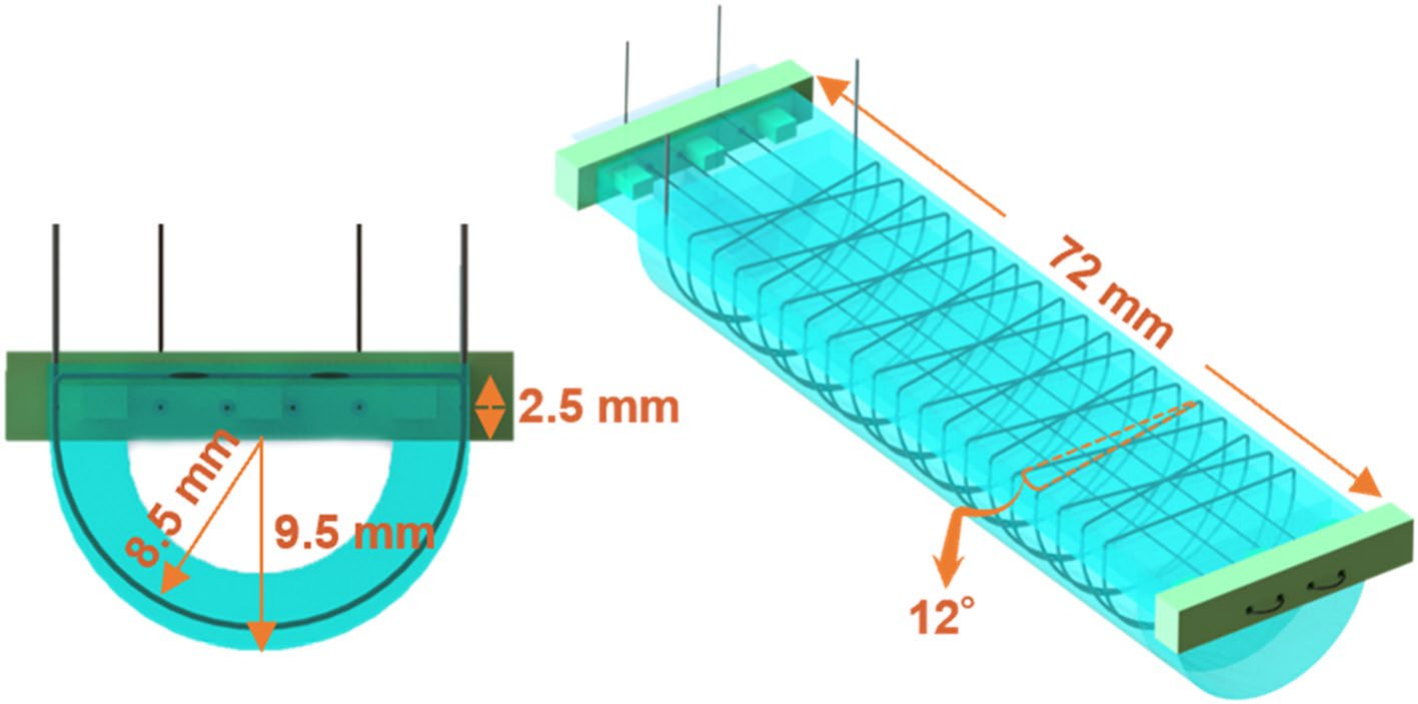
Dimensional parameters of the SMA wires integrated in the actuator. The wire is rounded twenty times around the actuator with a pitch of 6 mm and helix angle of 12 degree.

Upon functioning in a soft gripper or a bionic hand, damage can occur anywhere in the actuator, on both the top and the bottom side. Closing the incisions made at the top of the chamber is the sole responsibility of the radial wire, while both wires can assist in closure of damages in the bottom of the actuator (backbone). To investigate the limit in the size of the damages, here the transverse damages, which can be closed by SMA wires, the effective contraction length of the integrated wires along the chamber are calculated. The length of the longitudinal wire (288 mm) is equal to four times the length of the chamber (Fig. 6). However, the effective length of damage closure is one fourth of the entire length of the wire as it passes from the backbone of the actuator for four times (going and returning). Therefore, considering 4% of pre-straining, large damage of up to approximately 3 mm can be closed. For the radial one, the length is calculated as follows:

$$L= \sqrt{{P}^{2}+{C}^{2}}$$

where $L$ is length of a half-helix and $P$ and $C$ are the pitch and the circumference of the half-helix:

$$P= \frac{{P}_{full helix}}{2}=3 mm$$

$$C= 3.14\times 8.5=26.7 mm$$

$$L= \sqrt{{3}^{2}+{26.7}^{2}}=26.85 mm$$

Helixes are rounded twenty times around the chamber (Fig. 6). The total length of the helix and the total contraction along the length of the chamber by radial reinforcement is then calculated as follows:

$$L_{total} = \left\{ {26.85 \times 20} \right\} + \left\{ {2.5 \times 20} \right\} + \left\{ {17 \times \tan 12 \times 18} \right\} + \left\{ {17} \right\} = 719\,\,{\text{mm}}$$

$$L_{contractoin} = \left\{ {\left\{ {26.85 \times 10 \times \sin 12} \right\} + { }\left\{ {2.5 \times 10 \times \sin 0} \right\} + \left\{ {17 \times \tan 12 \times 9 \times \sin 12} \right\} + \left\{ {17 \times \sin 0} \right\}} \right\} \times 0.04 = 2.5\,\,{\text{mm}}$$

Based on the length of the longitudinal and radial wires and the data in Table S1, indicating the resistance and the recommended current, almost 5 V and 16 V is needed for their respective activation. When the radial wire is activated, the generated heat produced by the Joule effect is transferred to the backbone, also triggering the longitudinal SMA wire. Once this happens, the gap on the top side of the actuator opens further as the activated longitudinal wire bends the actuator for a few degrees. To prevent this issue, the high temperature SMA wire with 88 °C as the start activation temperature is employed for the backbone and the low temperature SMA wire for radial reinforcement (Table S1). As such, for closing a damage at the top side, 68–78 °C is needed which is below the start activation temperature of the high temperature wire (88 °C) (Table S1). To heal a damage at the bottom of the actuator, the straight wire is triggered, by rapidly heating it up to 88–98 °C for a short period of time to close the damage, which is followed by cooling down to about 75–80 °C for further healing, well below the gel transition of the self-healing polymer matrix (100 °C)^1^. Note that this healing temperature is above the relaxation temperature of the high temperature wire (72 °C), and consequently, it keeps its contraction during healing. The datasheet of the SMA wires is available in Table S1.

**Figure 7:**
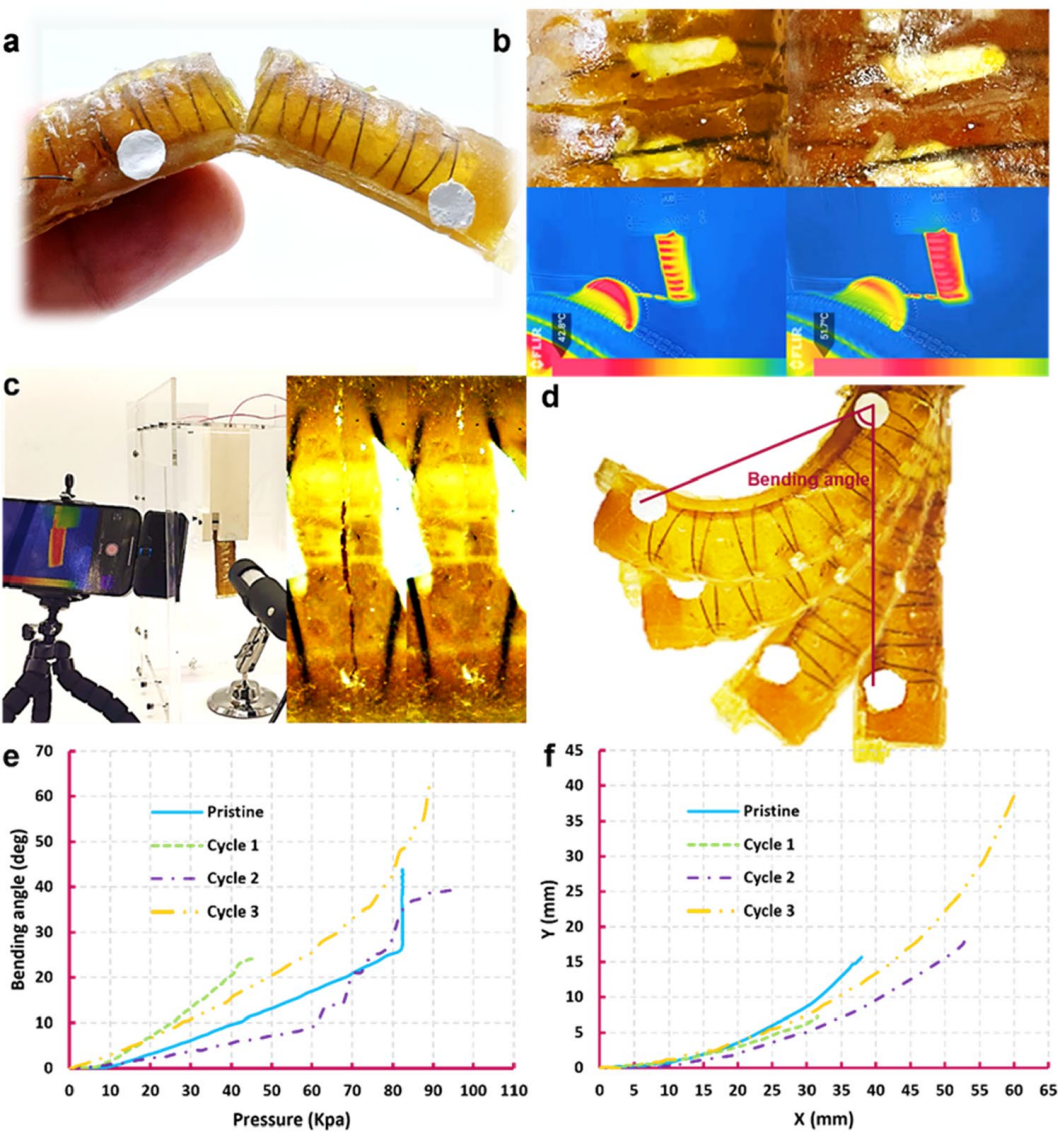
Damage closure and healing of the SMA reinforced soft healable actuator. The actuator was heated up for 45 min and kept at room temperature for one day. (**a**) Damaged SMA-reinforced actuator. (**b**) Closing the damage at the top side of the actuator. (**c**) Closing the damage at the backbone of the actuator. (**d**) Bending performance of the healed actuator. (**e**) Comparing the bending function of the actuator in multiple damage-healing cycles. (**f**) Comparing the fingertip trajectory of the actuator in multiple damage-healing cycles.

Figure 7 depicts the process of CTH in the bending actuator. To illustrate that healing also recovers the CTH property, three consecutive damage-healing cycles are studied. Each time the actuator is damaged at a new location (Movies S7 and S8). The fingertip trajectory and bending angle are compared before damage and after healing to show the ability of the system to recover its functionality. To do that, a linear pressure controller system inflates the chamber (Fig. S5), while the motion of the robot is recorded by a camera for further analysis (Sect. S8)^49^. Considering the viscoelastic property of the material and the stochastic behavior of soft robots, the robot is bent almost similarly in all the cycles (Fig. 7). We also observed in the actuator that without any manual intervention nor inflation of the chamber the SMA wires can again be contracted by Joule-effect heating for more than one damage-healing cycle, excluding the need for pre-straining of the embedded SMAs. The authors believe that it is the elastic response of the self-healing material that re-elongates the integrated SMA wires upon cooling down, straining them to allow for a subsequent CTH procedure and allowing for multiple damage-healing cycles. There is no need to disassemble the actuator from the station and take it to a rest position for healing. Taking advantage of the SMA-based CTH-approach, the robot performs in-situ closing and healing of large damages without the need of any external intervention. Furthermore, upon contraction of the wires, the entire chamber will be affected as the wires are embedded throughout the chamber. In rare instances where multiple significant damages occur in the actuator, the system can simultaneously close them. Clearly, intrinsic self-healing materials have the ability to simultaneously heal multiple damages, regardless of their location or number, as long as the sides of the damages are properly reconnected. Video evidence in supplementary material (Movies S9 and S10) demonstrates simultaneous multiple damage closing in the actuator. It is important to consider that synthetic materials do not have the ability to regenerate or grow. Therefore, if a portion of the material structure is removed, it cannot heal or repair itself without the replacement of that missing piece. This can also be seen in Movie S10.

It should be noted that 45 min for heating the actuator up and remaining at room temperature for one day was enough to recover the function of the actuator, although for a better recovery of the initial material properties we considered an hour for heating the non-reinforced specimens up and three days remaining at room temperature.

## Discussion

This paper presented major steps towards the autonomy of the damage healing ability of soft robots to cast away their dependence on human intervention in their healing procedure. A soft bending actuator was reinforced with SMA wires to integrate two assistive mechanisms needed for accelerated, efficient and autonomous healing. First, the shape memory effect of the SMA wires closes the damage, which enables the healing. This is an essential step in the healing action in all types of healable systems and has largely been overlooked in scientific literature. Second, heating up the wires synergistically triggers the healing via the Joule effect. These two assistive mechanisms improve the healing efficiency and rate, respectively. Using this SMA assistive healing, we presented for the first time a self-healing soft actuator that does not require human or external intervention to close and heal large damages. This is a major leap forward for self-healing robotic systems to surpass an important limitation in the healing capabilities known in natural systems.

Although we have provided an easy and integrated solution for autonomous damage closing and healing triggering of soft robots, with a broader vision, other integrated mechanisms which are not directly related to the healing procedure can still be included. It is true that the healing procedure can be settled into a routine. However, integration of a damage detection sensory network as the future work can take the healing action one step forward, to a higher level of autonomous healing. Moreover, for those self-healing soft robots that need to be thermally triggered for healing, integration of a temperature sensor in the system can increase the robustness of the robot in terms of autonomy from human intervention to be healed in various and dynamic working conditions. It should be noted that there is no need to keep the temperature of healing of the presented self-healing soft robot at an exact amount. Being able to control the temperature in a range of 5 °C higher and lower than the desired amount which is 80 °C is enough. The desired healing temperature can be much less in case of small damages and even until room temperature healing. The authors recommend and aim at integrating a Positive Temperature Coefficient material as the temperature sensor^7^. One of the other limitations of the system is in rare cases where a piece of the material of the system is completely removed. Clearly, the human assist is needed to replace the removed piece for the healing. Nevertheless, the integrated SMA wires reinforce the robot from another point of view, preventing the robot to be e.g., cut in two. The SMA wires are very strong and can very hardly be cut even with scissors.

### Potential applications of the technology

**Figure 8:**
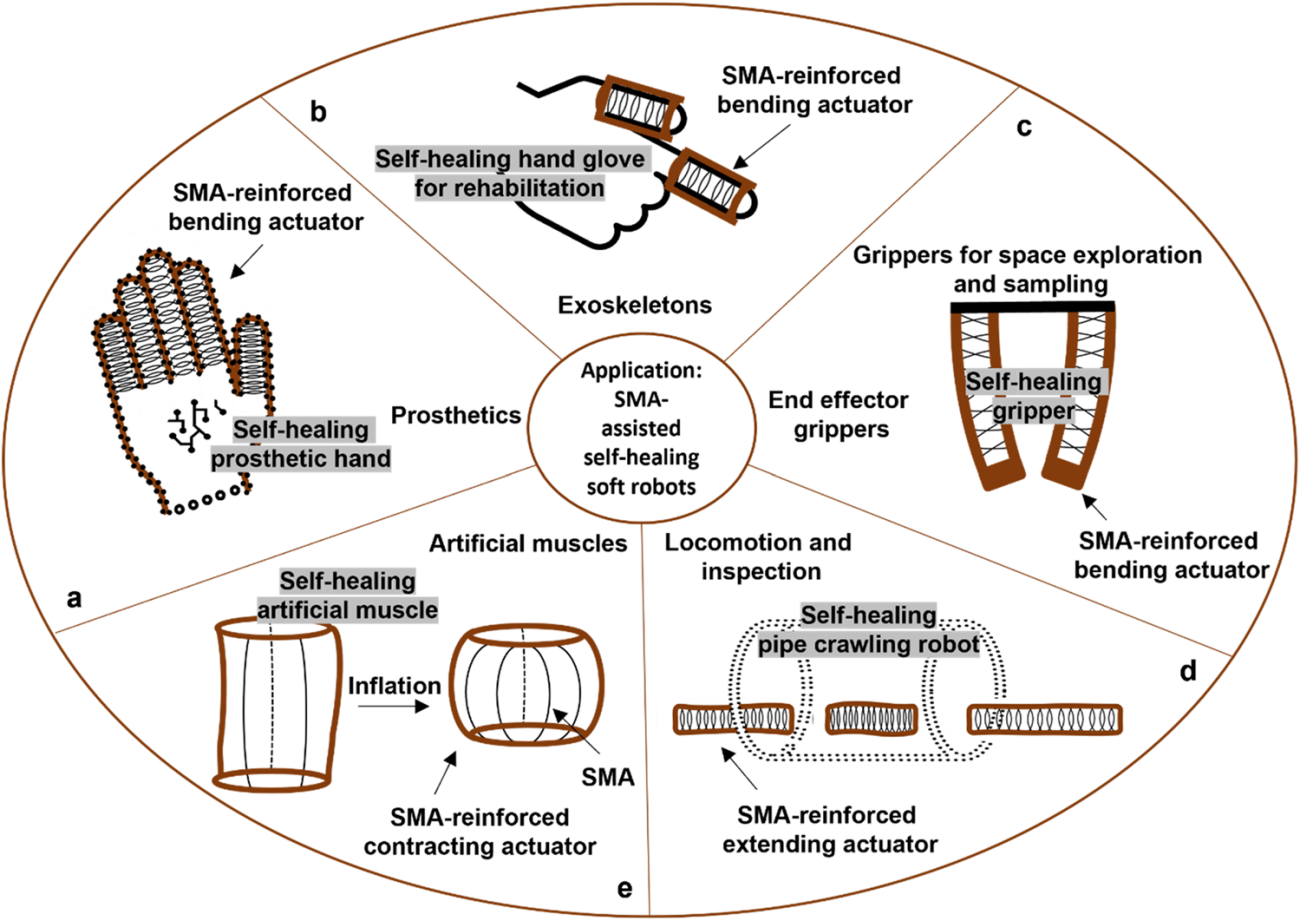
Some potential applications for the integration of the technology. (**a**) Fiber-reinforced bending actuators as fingers of a hand prosthesis. (**b**) Fiber-reinforced actuators used in a hand rehabilitative glove. (**c**) Soft grippers applied for space exploration and sampling can benefit from the technology. (**d**) A pipe-crawling robot made out of different fiber-reinforced actuators. (**e**) Pleated fiber-reinforced pneumatic artificial muscle. SMA wires can stretch along the creases instead of nylon fibers.

The proven concept can be applied to many of the current fiber-reinforced soft actuators, including bending actuators, extending actuators and artificial muscles^50^. By replacing the fibers with SMA wires and the soft matter with self-healing polymer material, the actuators are equipped with autonomous closing and healing ability. Figure 8 shows some potential applications where our technology can replace the current non-sustainable solutions by extending their lifetime. Although autonomous healing is in general economically and environmentally beneficial for a large variety of soft robotic applications, its impact in out-of-factory applications and for applications areas where humans have limited access is more significant. In these conditions, repair or replacement is highly costly or even impossible.

Upon damage in prosthetic hands (Fig. 8a)^51^, and hand exoskeletons (Fig. 8b)^52^, disassembly, damage realignment and external stimuli providing are demanding tasks that also need some equipment. Doing these actions, the users also need to meet with an expert. Nevertheless, Our integrated technology provides in situ healing and makes all the mentioned human interventions unnecessary. In addition, fiber-reinforced actuators are widely used in grippers. Grippers are usually in factories for sorting and manipulations. However, there are other very important applications out of the human access, e.g., robot manipulators for space exploration and sampling (Fig. 8c)^53^. Clearly, having self-healing grippers with autonomous healing of different damage sizes is of a great advantage, and probably essential.

The technology can also be used in production of fiber-reinforced extending actuators^52^. If in our presented design the longitudinal reinforcement is removed, the actuator will extend upon inflation. However, the radial reinforcement can preserve both damage closure and stimulation capabilities of the system. One of the outstanding applications of the extending actuators is locomotion for in-pipe or on-pipe inspection (Fig. 8d)^54^. Clearly, access to the robot while it is during the mission is not always possible. This is extremely critical and needs a robot with a high reliability factor, as suffering from a damage in the middle of the mission exacerbates the problem. Our technology can ensure mission accomplishment, at least in cases where a damage in the chamber disrupts the function. In addition to the extending actuators, fiber-reinforced artificial muscles can also benefit from our presented technology (Fig. 8e). Artificial muscles are contractile linear actuators inspired by human muscles^55^. They are employed in diverse applications, such as rehabilitative and assistive actuators^56^. The use of self-healing materials and SMA wires increase the longevity of the artificial muscles and make the muscle independent of human intervention in the healing practice.

Note that the mobile applications of the presented healing technology require a portable energy source to activate the SMA wires. The energy consumption can differ based on the healing time of the used self-healing material. In our case and based on the healing time of the presented SMA-reinforced soft actuator which is 45 min, the Joule-effect energy consumption for the activation of the radial and longitudinal wires are 21 W-hour and 2.27 W-hour*.* This can pose a challenge for some robotics applications, as carrying energy sources is a challenge for most untethered soft robots^57^. However, by large scaling, researchers have reported the development of an untethered crawling robot that is capable of carrying batteries^58^. Furthermore, soft robots, such as soft grippers, can be used as a tool for larger mobile robots like the Boston Dynamics 4-legged robots, where carrying energy sources is not an issue. Research into rechargeable energy sources that harness natural energy, such as sunlight, is also promising in this regard.

All in all, we have presented few potential applications where our demonstrated technology can be integrated and increase the level of sustainability, reliability and autonomy in soft robots. If self-healing is to be integrated in the future soft robotics applications, autonomous recontact of the fracture surfaces will be essential.

## Supplementary Information

Supplementary Video 1.

Supplementary Video 2.

Supplementary Video 3.

Supplementary Video 4.

Supplementary Video 5.

Supplementary Video 6.

Supplementary Video 7.

Supplementary Video 8.

Supplementary Video 9.

Supplementary Video 10.

Supplementary Information 1.

Supplementary Information 2.

## Data Availability

The data that support the findings of this study are available in the article and its supplementary information files. Any other information will be provided upon request.
