# Supplementary material for: Assisted damage closure and healing in soft robots by shape memory alloy wires
Source: Sci Rep. 2023 May 31;13:8820. doi: 10.1038/s41598-023-35943-6 (PMC10232441; doi:10.1038/s41598-023-35943-6)
Supplement: Supplementary file 11 — Supplementary Information 1. [file 41598_2023_35943_MOESM11_ESM.pdf]

**Supplementary material**

**Title:** Assisted damage closure and healing in soft robots by shape memory alloy wires

**Authors:**

Seyedreza Kashef Tabrizian,[1]* Seppe Terryn,[1,2] Aleix Costa Cornellá,[2] Joost Brancart,[1,2] Julie Legrand,[1] Guy Van Assche,[2] Bram Vanderborght[1]

**Affiliations:**

[1]Brubotics, Vrije Universiteit Brussel (VUB) and Imec; Brussels, Belgium.
[2]Physical Chemistry and Polymer Science (FYSC), Vrije Universiteit Brussel (VUB); Brussels, Belgium.

*Seyedreza.Kashef.Tabrizian@vub.be

## 1. Uniaxial tensile tests for material characterisation

Both materials were characterized by uniaxial tensile test performed on a TA Instruments DMA Q800. The tests were performed at room temperature. The specimens were in rectangular shape with a width and thickness of 5 mm and 1.5 mm. They were clamped between two film tension clamps with a 5mm distance in between while strained at a rate of 1% $s^{-1}$. The Young's modulus was extracted from the initial linear region (0-1% strain) of the stress-strain curve.

## 2. Processing of the SMA reinforced specimens

The specimens were made by casting the material into the 3D printed molds using a PRUSA SL1 3D printer. Two clamps were planted at the two ends of the specimens and in the molds. These SLA 3D printed clamps close the sides of the molds, help to fasten the SMA wires and distribute the contraction force of the SMA wires evenly and efficiently. The wires were embedded in the middle of the samples for a better heat distribution during heating/healing by Joule-effect. In fig. S1 all the specimens used for tensile test (fig. S1a), bending test (fig. S1b) and ultrasound test (fig. S1c) with their dimension are shown. . The bending test was done for three damage-healing cycles, whereas the tensile test was destructive and performed one time. Ultrasound test was done for one damage closure cycle.

## 3. Ultrasound test

Using an ultrasonic pulse analyser, damage closure was assessed in a much faster and easier method. However, it just shows how efficient the damage has been closed and cannot provide information about the restoration of mechanical properties of the composite. We used PULSONIC 58-E4900 and read the data with a frequency of 1 Hz. The position of the probes were fixed in all the experiments. Moreover, due to the softness of the specimen, it was placed on a manual lift platform to adjust the height and as such keeping constant the contact pressure between it and the probes in all experiments (fig. S2).

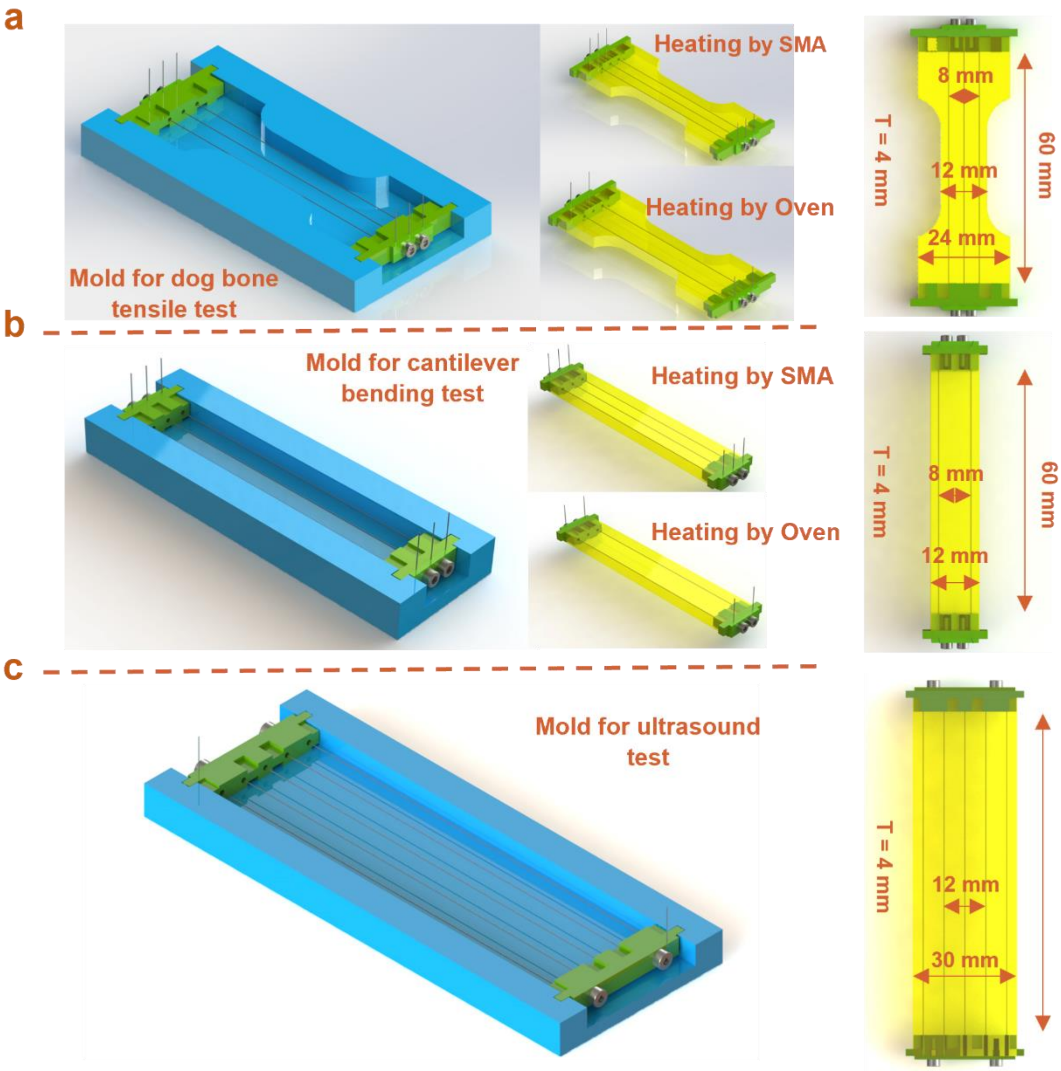


**Fig. S1. SMA-reinforced specimens.** They were fabricated by molding/casting the MA material into the 3D printed molds. (a) The specimens for tensile test. (b) The specimens for bending test. (c) The specimen for ultrasound test.

## 4. Finite element simulation of the bending actuator

The matrix (IT-material) shows hyperplastic behaviour. Its characterization is needed for the finite element simulation which is used to analyse the design parameters and the function of the system before processing[32,39]. We employed uniaxial tensile and compression test (fig. S3a) and fitted different consecutive laws on the data (fig. S3b). The Ogden model with strain energy potential order of one was employed to characterize the material due to a stable behaviour in full range of the strain data. The material coefficients were $\mu 1 = 11293.13 Pa$, $\alpha 1 = 6.21$ , and $D1 = 7.18E - 06\ Pa$.

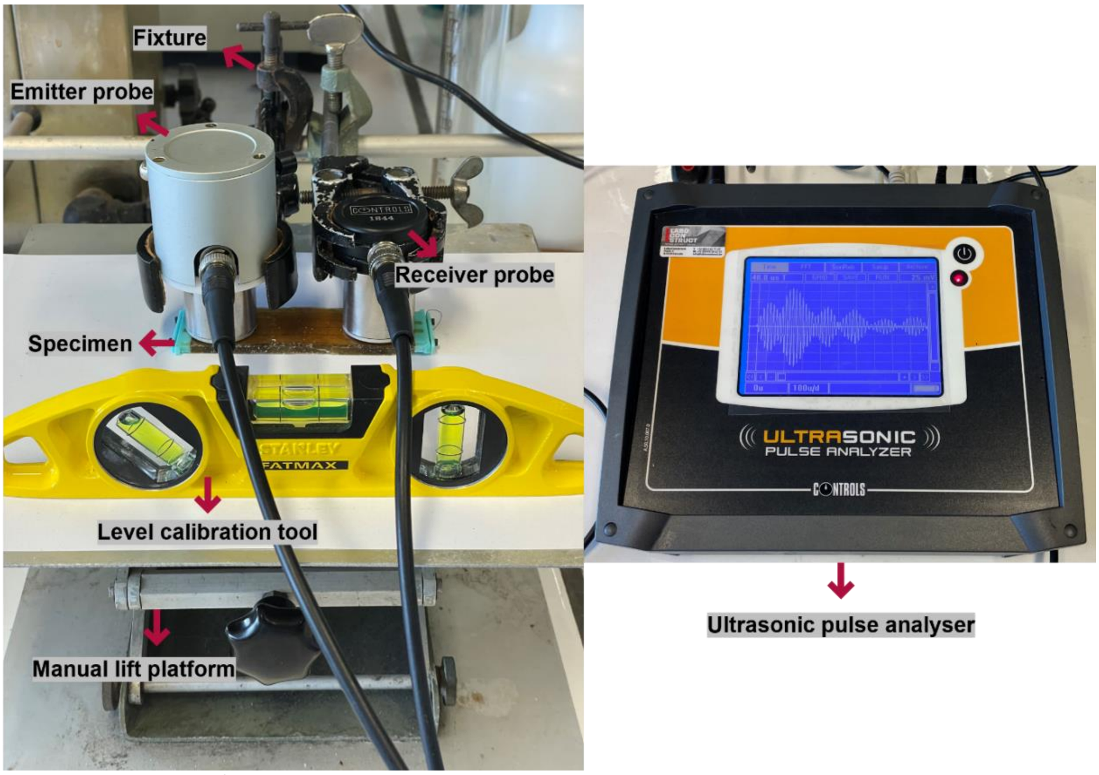


**Fig. S2. Test setup of the ultrasonic pulse analyser.**

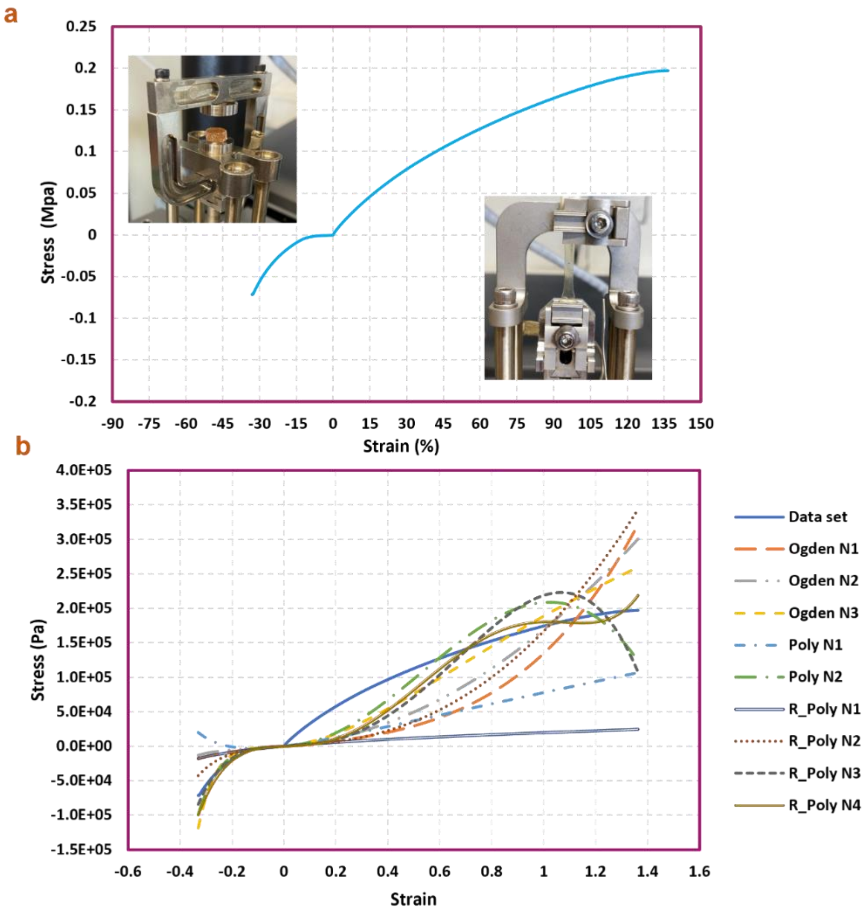


**Fig. S3. Hyper-elastic characterization of the soft material (itaconic-based material).** (a) The samples were under the application of uniaxial tensile and compression tests which are the least data needed for characterization. (b) Among various models, Ogden model with order 1 was used for characterization due to stability in all the strain range of the test data.

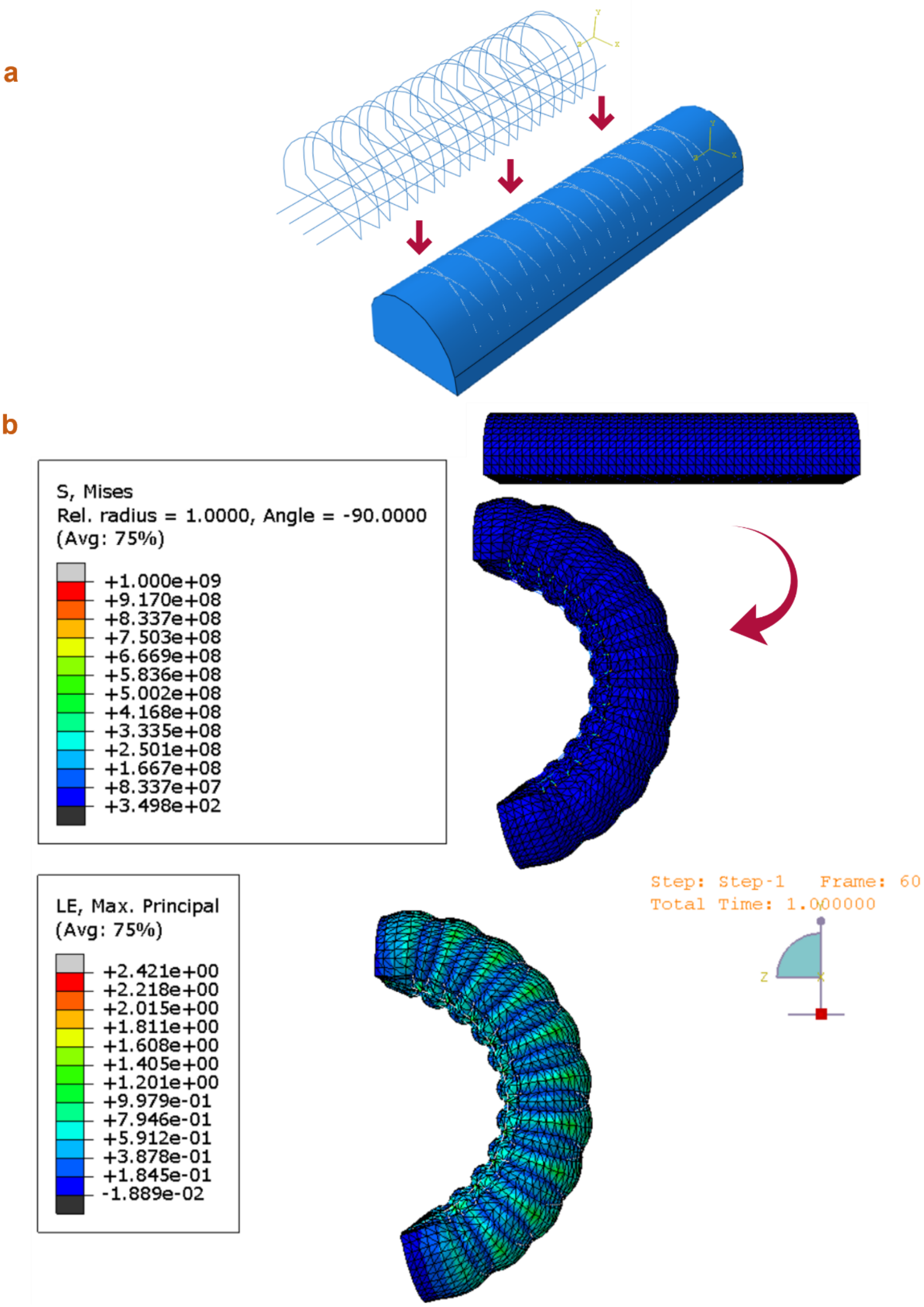


**Fig. S4. Finite element simulation of the actuator.** (a) The SMA wires were drawn in the Abaqus while the chamber was imported. Thereafter, they were assembled. (b) Bending performance of the actuator under 40Kpa pressure.

Note that this hyperplastic characterization was done for the softer material (IT material) used to make the actuator out of. All the tests were done using TA Instruments DMA Q800 with the appropriate clamp for each. The Poisson's ratio of the material was considered to be 0.5, similar to most of the elastomers. Density of the material was 1200 $kg/m^3$. SMA wires were modelled as elastic material with the properties available in table S1. The chamber was imported to the environment of the Abaqus while the wires were drawn in the software and then assembled with the chamber as seen in fig. S4a. A

tie constraint was defined between the wires and the outer surface of the chamber. The pressure was applied to the inner surface of the chamber and one side of the chamber was fully constrained as the boundary condition. The element of the SMA wire was B32 whereas the matrix was defined by C3D10H. As seen in fig. S4b, the actuator reached to almost 90° of bending angle while inflated with 40 Kpa. In the experiments, more than 80 Kpa was needed to reach to almost the same level of bending angle. This clear difference originates from two main reasons. First, uniaxial hyperplastic characterization of the materials cannot exactly mimic the properties of it. In the future, biaxial tensile test can be performed in case of the availability of the equipment. Second, and most importantly, is the viscoelastic property of the material. In different temperature of the working environment, the properties of the material may change.

## 5. Processing of the SMA reinforced actuator

Fiber-reinforced pneumatic actuators are well-known designs among soft robotics engineers[31-33]. Usually, bending, twisting or extension is created in a cylindrical or half-cylindrical actuators composed out of flexible elastomeric material by embedding a combination of radially rounded fibers and a strain-limiting layer that act as reinforcements. Among several existing applications, we have selected a bending actuator to demonstrate our technology in a soft robotic application (Fig. 1). One long SMA wire is winded in helical configuration around the half-cylindrical actuator, restricting expansion of the flexible polymers in the radial direction, preventing ballooning and enhancing bending. The SMA wire is winded clockwise and anti-clockwise around the actuator. This symmetrical reinforcement allows for higher contraction and closure of incisions with any random orientation. In addition, another SMA wire is positioned at the bottom layer, preventing elongation in the bottom plane and therefore enhancing the bending motion. Figure 2 shows the processing steps of the actuator. There is a main mold (3D-printed by SLA technique) for each of the first two steps that are sectioned from the middle and then bolted together (mold N1 and mold N2). First, the half-cylinder rod which is spray coated by a release agent is positioned in the mold N1 and then the side clamps are placed. The longitudinal SMA wire goes through the holes of the clamps round and round. Finally, the material is poured into the mold N1 and is cured for one day at room temperature.

As seen, the path for the radial reinforcement is considered in mold N1 for the ease and equally distancing (important to prevent ballooning) of wire twisting in the next step. In addition, this ensures that the wires are completely embedded in the actuator for an efficient force transmission during damage closure. The pitch for radial reinforcement is 6 mm. Based on the bending simulation by a finite element analysis (FEA) that simulates the bending motion (fig. S4) and heating experiments monitored by the thermal camera, this provides the adequate reinforcement needed for actuation and a fairly homogeneous heat distribution throughout the actuator. In the FEA simulation the IT-material is modelled using a hyper-elastic constitutive law, the Ogden model (fig. S3)[32,39].

Next, the final merged part, containing the half-cylinder covered with cured material around, the planted clamps and the embedded longitudinal SMA wire, is demolded and equipped with the radial reinforcement. To prevent short circuit in the wire, teflon tape is used where the wire passes over itself. Thereafter, the part in mold N2 is coated by pouring more of the of IT-material into the mold, covering and embedding the radial SMA reinforcements. After one day at ambient conditions, the part is demolded. Finally, the half-cylinder rod is pulled out from the chamber after which it is equipped with the

inlet and is sealed by self-healing, through local heating of the material. For longitudinal reinforcement high temperature (HT) SMA wire with a diameter of 0.2 mm and for radial one LT SMA wire with 0.3 mm in diameter are used (table. S1).

## 6. Mechanical tests on the SMA-reinforced specimens

Rectangular composite specimens (fig. S1) were put under the application of cantilever bending test. TA Instrument Q800 DMA machine was used. The distance between the clamps were 41 mm and the specimens were loaded with a rate of 20 % per minutes until the full range of the machine (they reached to almost 22% strain). Using the cantilever clamps prevent slippage of the specimen in bending test which can occur due to high flexibility and low flexural strength of the composite. For tensile tests, dogbone composite specimens were stretched using Tinius Olsen tension machine with a rate of 30 %. $S^{-1}$ until fracture.

## 7. Linear pressure controller system

Figure S5 shows a top view of the linear pressure controller system. The outlet of the 60 CC syringe goes to the actuator and the Honeywell pressure sensor (SSCDANN100PGAA5) via a male tee connector. The syringe can be easily replaced by a 200 cc one for larger actuators or applying more pressure. The plunger of the syringe was attached to the nut by a 3D-printed interface. The nut moves by the rotation of the leadscrew (Tr 12×3) which was coupled to the bipolar NEMA17 stepper motor via Ruland aluminium flexible beam coupling to compensate for the probable misalignments. The motor was controlled using a driver (TMC 2130 V1.1 SPI 1/256 Microsteps) programmed by an Arduino Uno ATmega328P. The logic voltage is 5V while the power supply of the stepper motor is 120 W, 24 V.

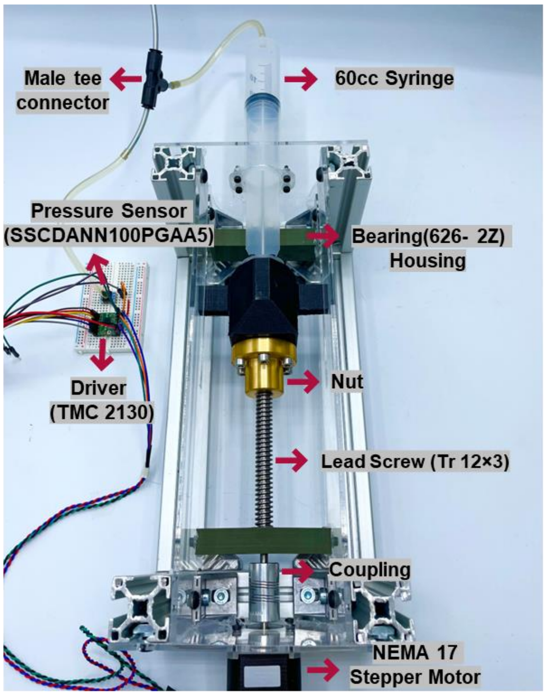


**Fig. S5. The linear pressure controller system used to actuate the finger.**

## 8. Tracking the motion of the actuator

Two white round markers were attached to the finger. One to the base of the finger near to the inlet and one to the fingertip. While actuated, the bending motion of the finger was recorded by a phone camera. The videos were processed in Matlab (The Mathworks, Massachussets, US) using *imfindcircles* function to get the position of the markers during time. This algorithm was previously tested on a mechanical micrometer device and showed an accuracy of 0.09 mm in predicting the position of the markers[43].

## 9. SMA wires

Flexinol muscle wires from Dynalloy with 0.2 and 0.3 mm in diameter, made out of highly processed strands of Nickle-Titanium alloy Nitinol are used. They are in two different start transition temperatures called low temperature (LT) and high temperature (HT) that are 68 °C and 88 °C, respectively. They have been pre-strained by 4 % upon delivery. The datasheet of the SMA wires are in table S1. For all the specimens, LT activation wires with 0.2 mm in diameter are used while in the actuator a combination of the wires are embedded which will be clarified in the corresponding section.

**Table S1. Properties of the SMA wires**

<table>
<tr><th></th><th>Diameter (mm)</th><th>0.2</th><th>0.3</th></tr>
<tr><td rowspan="2">Physical</td><td>Linear resistance (Ω/m)</td><td>31</td><td>13</td></tr>
<tr><td>Recommended current (mA)</td><td>610</td><td>1.750</td></tr>
<tr><td rowspan="5">Thermal</td><td>Type</td><td>LT Alloy</td><td>HT Alloy</td></tr>
<tr><td>Activation start temp (°C)</td><td>68</td><td>88</td></tr>
<tr><td>Activation finish temp (°C)</td><td>78</td><td>98</td></tr>
<tr><td>Relaxation start temp (°C)</td><td>52</td><td>72</td></tr>
<tr><td>Relaxation finish temp (°C)</td><td>42</td><td>62</td></tr>
<tr><td rowspan="3">Phase related</td><td>Phase</td><td>Martensite</td><td>Austenite</td></tr>
<tr><td>Resistivity (μΩcm)</td><td>76</td><td>82</td></tr>
<tr><td>Young's modulus (Gpa)</td><td>28</td><td>75</td></tr>
<tr><td rowspan="6">Material</td><td>Max recovery force (Mpa)</td><td colspan="2">600</td></tr>
<tr><td>Rec deformation force (Mpa)</td><td colspan="2">35</td></tr>
<tr><td>Breaking strength (Mpa)</td><td colspan="2">1000</td></tr>
<tr><td>Density (g/cc)</td><td colspan="2">6.45</td></tr>
<tr><td>Poisson's ratio</td><td colspan="2">0.33</td></tr>
<tr><td>Rec deformation ratio (%)</td><td colspan="2">3-5</td></tr>
</table>
