# Supplementary material for: Assisted damage closure and healing in soft robots by shape memory alloy wires
Source: Sci Rep. 2023 May 31;13:8820. doi: 10.1038/s41598-023-35943-6 (PMC10232441; doi:10.1038/s41598-023-35943-6)
Supplement: Supplementary file 12 — Supplementary Information 2. [file 41598_2023_35943_MOESM12_ESM.docx]

Movie S1: Summary of the research.

Movie S2: Damage closure in an oven.

Movie S3: Damage closure by Joule-effect.

Movie S4: Damage creation in the specimen.

Movie S5: Tensile test.

Movie S6: Bending test.

Movie S7: Damage closure in top side of the actuator by Joule-effect.

Movie S8: Damage closure in the bottom side of the actuator by Joule-effect.

Movie S9: Simultaneous multiple damage closure in the top side of the actuator by Joule-effect.

Movie S10: Simultaneous multiple damage closure in the bottom side of the actuator by Joule-effect.
